# Cryoprotectants and Extreme Freeze Tolerance in a Subarctic Population of the Wood Frog

**DOI:** 10.1371/journal.pone.0117234

**Published:** 2015-02-17

**Authors:** Jon P. Costanzo, Alice M. Reynolds, M. Clara F. do Amaral, Andrew J. Rosendale, Richard E. Lee

**Affiliations:** Department of Zoology, Miami University, Oxford, Ohio, United States of America; University of Sao Paulo, BRAZIL

## Abstract

Wood frogs (*Rana sylvatica*) exhibit marked geographic variation in freeze tolerance, with subarctic populations tolerating experimental freezing to temperatures at least 10-13 degrees Celsius below the lethal limits for conspecifics from more temperate locales. We determined how seasonal responses enhance the cryoprotectant system in these northern frogs, and also investigated their physiological responses to somatic freezing at extreme temperatures. Alaskan frogs collected in late summer had plasma urea levels near 10 μmol ml^-1^, but this level rose during preparation for winter to 85.5 ± 2.9 μmol ml^-1^ (mean ± SEM) in frogs that remained fully hydrated, and to 186.9 ± 12.4 μmol ml^-1^ in frogs held under a restricted moisture regime. An osmolality gap indicated that the plasma of winter-conditioned frogs contained an as yet unidentified osmolyte(s) that contributed about 75 mOsmol kg^-1^ to total osmotic pressure. Experimental freezing to –8°C, either directly or following three cycles of freezing/thawing between –4 and 0°C, or –16°C increased the liver’s synthesis of glucose and, to a lesser extent, urea. Concomitantly, organs shed up to one-half (skeletal muscle) or two-thirds (liver) of their water, with cryoprotectant in the remaining fluid reaching concentrations as high as 0.2 and 2.1 M, respectively. Freeze/thaw cycling, which was readily survived by winter-conditioned frogs, greatly increased hepatic glycogenolysis and delivery of glucose (but not urea) to skeletal muscle. We conclude that cryoprotectant accrual in anticipation of and in response to freezing have been greatly enhanced and contribute to extreme freeze tolerance in northern *R. sylvatica*.

## Introduction

Although freeze tolerance in vertebrates was first reported over 30 years ago [1] and dozens of papers on the subject have since appeared, relatively little is known about variation in freeze tolerance capacity among populations of conspecifics [2–5]. We recently described the extraordinary capacity for freeze tolerance in a subarctic population of the wood frog (*Rana sylvatica*), a North American species that ranges from the southern Appalachians to within the Arctic Circle. Whereas frogs indigenous to the upper Midwestern United States and southern Canada tolerate freezing only to temperatures as low as -3 to -6°C, frogs from Interior Alaska readily survived experimental freezing to -16°C [6].

We hypothesize that extreme freeze tolerance in this northern phenotype derives in part from an enhanced cryoprotectant system that uses urea, which accumulates in autumn and early winter, before freezing occurs, and glucose, which is quickly mobilized from liver glycogen after freezing begins. Both agents colligatively reduce the amount of ice forming in the body, but each also serves other functions that enable cells and tissues to withstand myriad stresses caused by freezing and thawing [7].

Urea accumulation is a universal amphibian response to osmotic challenge, such as dehydration or salt acclimation, which serves to limit the transcutaneous loss of body water [8]. Absent osmotic stress, uremic levels remain low, typically only 5–10 μmol ml^-1^ [9]. However, during physiological preparation for winter, plasma urea accrues to high levels (>100 μmol ml^-1^) in Alaskan *R*. *sylvatica* kept hydrated under humid conditions [6], raising questions about the underpinnings of nitrogen metabolism in these frogs. We found that enhanced ureagenesis is subsidized by muscle protein, but we did not ascertain whether the catabolism is a regulated process or simply a manifestation of fasting during winter conditioning. In addition, Alaskan frogs—but not frogs from a temperate population—co-accumulate with urea a significant quantity (~73 μmol ml^-1^ plasma) of an osmolyte(s) whose identity was not determined.

The capacity for freezing survival in *R*. *sylvatica* is strongly influenced by the quantity of glucose mobilized during freezing. In Alaskan frogs, glycemia rose to 217 μmol ml^-1^ during a 48-h bout of experimental freezing to -2.5°C [6]. However, because they mobilized only a portion of their hepatic glycogen store, we conjectured that they synthesize even more glucose in liver if the freezing exposure is protracted or particularly severe. This is an important consideration because, in Interior Alaska, frogs must survive extended bouts of freezing with hibernaculum temperatures reaching minima of -9 to -18°C [10]. Furthermore, because these frogs remain hyperglycemic many days after thawing [6], individuals undergoing multiple freeze/thaw cycles potentially can accrue higher levels of glucose than can be achieved by frogs experiencing a single freezing episode.

This work provides new information about the cryoprotective systems supporting extreme freeze tolerance in a subarctic population of *R*. *sylvatica*. We report that urea accrual in frogs preparing for hibernation is subsidized by regulated proteolysis in muscle, a response strongly influenced by water balance. In addition, we demonstrate that glucose mobilization from hepatic glycogen reserves is responsive to severity of the freezing episode, and that multiple freeze-thaw cycles, while not essential to high glucose mobilization, improve the distribution of cryoprotectant to peripheral tissues.

## Materials and Methods

### Ethics Statement

We collected post-metamorphic *R*. *sylvatica* by hand or dip net from publicly-owned woodlands in Fairbanks North Star Borough, Alaska, USA (64.8°N, 147.7°W), between 30 July and 5 August, 2012, under approved methods and appropriate permits issued by the Alaskan Department of Fish and Game. Protocols for experimentation and euthanasia, which was carried out by double-pithing without the use of anesthesia, were approved by the Institutional Animal Care and Use Committee of Miami University (Research Protocol #812).

## Experimental Animals and Winter Conditioning

After their capture, frogs were topically treated with tetracycline HCl, placed in plastic cups containing a moist sponge, and shipped under refrigeration to our laboratory, where we removed the sponge and added fresh tetracycline solution. They were kept at ~18°C until the next day, when we transferred each frog to a translucent, polyethylene container (floor area, 56.7 cm^2^) that was closed with a perforated lid. Each such cage contained a shelter (an inverted, opaque cup with an opening in its side) that rested on two layers of unbleached paper towel, which we replaced at 1-week intervals. Except as noted elsewhere, we wetted this substratum with 2.5 ml dechlorinated water, which thoroughly dampened the fibers and left a thin film of water on its surface.

Following Costanzo et al. [6], we promptly initiated a winter-conditioning regimen that gradually induced frogs into dormancy, using a programmable environmental chamber (Percival, model I-35X; Boone, IA, USA) to expose them to dynamic, diel cycles of temperature and full-spectrum lighting over a 5.5-week period. Thermal and photic conditions were based on climatological records obtained from the National Oceanic and Atmospheric Administration’s National Climatic Data Center for an area near the collection site. During the first week, temperature in the chamber varied daily from 17.0 to 8.0°C and the photophase was 16.5 h. We made decremental adjustments weekly such that, during the final week, in mid-September, temperature varied daily from 13.0 to 2.5°C and the photophase was 13.3 h. We kept the frogs under these latter conditions for an additional 3 d before transferring them to a darkened cold room (4°C) where they were sampled immediately (*Water Balance Experiment*; see below) or held until used in November (*Freezing Experiment*; see below).

During winter conditioning, frogs were fed *ad libitum* with small (~13 mm) crickets that had been dusted with a vitamin supplement (ReptoCal, Tetrafauna, Blacksburg, VA, USA). On three days each week we added as many as five live crickets (actual quantity depending on recent feeding history) and removed any dead crickets from the cages. Some frogs fed more readily than others, but most refused food after the last week in August and so the feedings were discontinued. A few frogs appeared ill or died and were eliminated from the study; we collected data only from healthy individuals.

## Water Balance Experiment

We investigated the effect of hydration state on various physiological adjustments accompanying physiological preparation for winter. To provide sufficient tissue for the analyses, we used the larger of the frogs available for study; for this pool, standard body mass (SBM), determined by weighing frogs after removing any bladder fluid with a cloacal catheter, was 9.3 ± 0.4 g (mean ± SEM; range, 7–15 g; *N* = 32). We assigned each individual to one of three groups: late summer, winter-conditioned/hydrated (HYD), or winter-conditioned/dehydrated (DHYD). Assignments were made in a manner that standardized the SBM mean and variance among groups, but without regard to gender, which could be determined only upon dissection; sex ratio for the pool was 1.5:1.0 (male:female).

Late-summer frogs (*N* = 8) were euthanized and sampled on the day after their arrival in our laboratory, whereas HYD frogs (*N* = 10) and DHYD frogs (*N* = 14) were first subjected to the full winter-conditioning regimen, as described above. HYD and DHYD groups were treated identically except that the latter was exposed to drier conditions. Initially, the substratum in cages with DHYD frogs received only 1.0 ml water, which dampened the paper and humidified the air, but did not provide a free-water surface. When replacing the substratum at weekly intervals, we reduced this amount to 0.5 ml (weeks 2–4), and added no water during the final week. By contrast, HYD frogs were continuously exposed to moist substratum throughout the regimen. We totaled the number of crickets consumed by each frog in both groups. Following winter conditioning, we euthanized all frogs and sampled their tissues as described in the next section.

## Morphometrics and Physiological Assays

Working inside a refrigerated (4°C) room, we determined the SBM of each frog, euthanized it by double-pithing, measured its snout-ischium length, and dissected it to expose the visceral organs. Blood was drawn into heparinized microcapillary tubes from an incision in the aortic trunk and centrifuged (2000 *g*, ~5 min) to isolate the plasma, which was promptly frozen in liquid N_2_. We quickly excised the intact liver, which was lightly blotted on laboratory tissue and weighed on an electronic balance. We also excised two muscles, gastrocnemius and gracilis, from the right hindlimb.

Portions of the liver and gracilis were immediately frozen in liquid N_2_; these samples, and the plasma, were stored at -80°C before metabolite assays were carried out. Additional portions of the liver and gracilis, plus the intact gastrocnemius, were blotted to remove extraneous moisture, weighed, placed in a 65°C oven, and reweighed when thoroughly dry. We determined initial water concentration in these samples by dividing the mass lost during drying by the mass of the dried tissue. From its water concentration, we estimated the mass of the entire, dry liver and, in turn, used this value to compute the hepatosomatic index (HSI, g dry liver g^-1^ dry body × 100). We removed, weighed, and discarded any coelomic fat body. The carcass was then weighed and thoroughly dried so that its water concentration could be determined and, by extrapolation, mass of the entire dry body could be estimated. We measured the length of the tibiofibula of the right hindlimb with dial calipers, this value being used to normalize the mass of the dried gastrocnemius to body size.

Dried carcasses (which lacked the fat body and liver, and some muscle tissue) were homogenized in a coffee grinder and then pulverized to fine granules with a mortar and pestle. We estimated organic concentration in these samples by burning a weighed aliquot (~125 mg) at 550°C for ~18 h in a muffle furnace (Thermolyne 48000, Waltham, MA, USA) and then reweighing the ash residue. Nitrogen and caloric concentrations were determined via the combustion method and bomb calorimetry, respectively, which were carried out under contractual agreement with the University of Arkansas Poultry Science Central Analytical Laboratory (Fayetteville, AR, USA). Total amounts of organic matter, nitrogen, and energy in carcasses were found by multiplying the sample concentrations by the dry body mass. Using the aforementioned procedures, we determined quantities of water, organic matter, nitrogen, and energy in a homogenized sample of the vitamin-fortified crickets (*N* = 50) used to feed frogs during winter conditioning.

We prepared deproteinized extracts of liver and muscle (gracilis) by homogenizing frozen samples in cold 7% (w/v) perchloric acid and then neutralizing the aqueous portion of the homogenate with potassium hydroxide. Extracts were assayed for urea, glucose, and lactate using urease, glucose oxidase, and lactate oxidase procedures (Pointe Scientific, Canton, MI, USA), respectively; metabolite concentrations were expressed as μmol g^-1^ dry tissue. Extracts were also assayed for glycogen in the following manner: a 100-μl aliquot of neutralized extract was incubated with amyloglucosidase (1 mg ml^-1^) in a 0.2 M sodium acetate buffer (pH 4.8; 40°C) and, after 2 h, the reaction was stopped by adding cold 7% (w/v) perchloric acid. Glucose was determined by glucose oxidase assay and the quantity generated during enzymatic digestion (i.e., after subtracting initial free glucose) equated to glycogen concentration, expressed as glucosyl units (μmol g^-1^ dry tissue). We computed hepatic glycogen content (μmol) as the product of the tissue concentration and dry organ mass, and glycogen richness (μmol g^-1^ dry tissue) by dividing hepatic glycogen content by dry body mass.

We analyzed the plasma for the three metabolites mentioned above, as well as total protein, which was measured using the Bradford method (BioRad; Hercules, CA, USA) with bovine serum albumin as the standard. Plasma osmolality was measured by freezing point-depression osmometry (Advanced Instruments, model 3320, Norwood, MA, USA) using appropriate sodium chloride standards.

Analysis of the free amino acid pool in late-summer, HYD, and DHYD frogs was made on samples prepared by combining similar quantities (55–70 μl) of plasma taken, as available, from all or most individuals within each group. The resultant plasma pools were thoroughly mixed, aliquoted into small portions, and frozen (–80°C) prior to further analysis. Amino acid quantitation was carried out using ion-exchange chromatography under contractual agreement with Biochemical Genetics Laboratory, Children’s Hospital Colorado (Denver, CO, USA).

## Freezing Experiment

In mid-November, we investigated certain physiological responses of hibernating frogs (SBM, 3.51 ± 0.15 g; *N* = 33; sex ratio, 1.2:1.0 male:female) to experimental freezing using a protocol that ensured frogs froze slowly, a condition that facilitates cryoprotective responses and improves freezing survival, and is ecologically relevant [11]. Frogs were housed individually inside a polyethylene container (“cage,” as described previously) on a substratum of moist paper towel. We covered each frog with an inverted cup (“shelter,” as described previously, but lacking the opening), which limited their movement and facilitated their extraction following freezing, and filled the remaining space with damp moss, which humidified the air and moderated the rate of cooling. We added ice chips which, upon cooling, served to initiate freezing of the moss and substratum and, ultimately, the frog. Containers were closed with a perforated lid and arrayed inside one of several opaque plastic boxes (0.0162 m^3^), which were covered and loaded into a programmable incubator (Percival, model I-35X, Boone, IA, USA) set at 0°C. Temperature was continuously recorded using several microprocessor-based loggers (Tidbit, Onset Computer Corporation; Pocasset, MA, USA) placed within a sham container (lacking a frog) inside each box and on shelves inside the incubator.

In one trial, we cooled frogs (*N* = 7) at a uniform rate (0.05°C h^-1^) to -8°C, a process requiring 160 h, and held them at that temperature for 6 h before dissecting them. In another, we cooled frogs (*N* = 7) at the same rate to -16°C, a process requiring 320 h, and held them at that temperature for 14 h before dissecting them. We subjected additional frogs (*N* = 12) to multiple freeze/thaw cycles. Parameters of this experiment were similar to those of the -8°C freezing trial, except that we interrupted cooling when the frogs had reached -4°C and raised the incubator temperature to 4°C. After the boxes had remained at 4°C for 12 h, during which time the frogs had visibly thawed but the ice surrounding them had not completely melted, we reset the incubator to initiate another cooling bout. After three such cycles, frogs were cooled to and held at -8°C for 5 h, following which some individuals (*N* = 7) were immediately euthanized and dissected; others (*N* = 5) were thawed at 4°C and monitored for signs of recovery, including exhibition of the “righting reflex” within 2 s of being placed on the dorsum. This multiple freeze/thaw trial lasted 19 d, with frogs experiencing a cumulative 15 d of freezing, assuming they were frozen whilst temperature was ≤ –0.7°C, the approximate freezing point of their body fluids.

Frozen frogs destined for physiological analyses, along with a set of reference frogs (*N* = 7) taken directly from their cages at 4°C, were quickly euthanized, dissected, and processed as described in *Morphometrics and Physiological Assays* (above), except that blood could not be collected from frozen frogs and no analyses were made on dried carcasses.

## Statistical Inferences

Summary statistics are presented as mean ± 1 SEM. We compared data from two groups using a Student’s *t*-test or, if not normally distributed, a Mann-Whitney *U*-test. We used an Analysis of Variance (ANOVA), followed by Student-Newman-Keul’s Multiple Comparisons Test, to compare data from three groups; data were first log transformed, as necessary, to meet assumptions of normality and homoscedasticity. In the few cases where transformation was ineffectual we used the nonparametric Kruskal-Wallis *H*-test, followed by a Dunn’s test, to compare values among groups. Significance was judged at *P*<0.05.

## Results

### Responses to Winter Conditioning

Late-summer frogs, which were dissected promptly after their arrival in our laboratory, had fed recently before their capture, as their guts contained arthropods in various stages of digestion. Frogs subjected to winter conditioning each consumed up to 19 crickets, with much of the feeding occurring earlier in the regimen, when ambient temperatures were higher. There was no difference (Mann-Whitney *U* = 69.5, *P*>0.99) in the numbers of crickets consumed by HYD frogs (5.4 ± 1.1) and DHYD frogs (6.0 ± 1.4). On average, each cricket weighed 151.6 mg (fresh mass) and contained 114.3 mg water, 4.21 mg nitrogen, and 0.837 kJ energy.

Tracking masses of individual frogs showed that all except one weighed slightly less following winter conditioning than they did initially. However, the measured reduction in SBM was greater (Mann-Whitney *U* = 17.5, *P* = 0.002) in DHYD frogs (15.3 ± 1.8%) than in HYD frogs (6.7 ± 1.6%). Accordingly, water concentrations in tissues of DHYD frogs were consistently lower than those in HYD frogs, although the differences were modest, being only 9% for liver, 16–19% for skeletal muscles, and 14% for carcass (Table 1).

**Table 1:** Water concentration (g water g^-1^ dry tissue) in select organs and carcass of *R*. *sylvatica* after winter conditioning, with or without moisture restriction.

	Hydrated	Dehydrated	P
Liver	2.04 ± 0.02	1.86 ± 0.02	<0.0001
Gastrocnemius	4.12 ± 0.08	3.45 ± 0.05	<0.0001
Gracilis	4.40 ± 0.10	3.58 ± 0.05	<0.0001
Carcass	3.57 ± 0.13	3.08 ± 0.07	0.0019
N	10	14	

Values are mean ± SEM.

Winter-conditioned HYD and DHYD frogs grossly resembled the late-summer frogs; indeed, there were no statistically significant differences among the three groups in snout-ischium length, body mass index, or even SBM (Table 2). Nevertheless, winter conditioning induced change in certain somatic variables and energy status. Notably, the substantial fat body of late-summer frogs was virtually absent in HYD and DHYD frogs. In addition, relative to late-summer frogs, carcasses of winter-conditioned frogs had lower concentrations of organic matter (4%) and energy (8–11%), albeit higher concentrations of nitrogen (7–14%); however, absolute amounts of these components did not vary among the groups. Furthermore, mass of the liver, as represented by HSI, was ~1.3-fold higher in winter-conditioned frogs (Table 2). This rise stemmed from glycogen storage, as the hepatic glycogen depot of DHYD frogs was nearly 50% greater than that of late-summer frogs; HYD frogs also had large glycogen depots, but the contrast with late-summer frogs was not quite significant. Glucose levels in liver were modestly, albeit significantly, higher in winter-conditioned frogs as compared to late-summer frogs.

**Table 2:** Somatic variables of *R*. *sylvatica* sampled before or after winter conditioning, with or without moisture restriction.

		Late summer	Hydrated	Dehydrated	P
Snout-ischium length (cm)		4.6 ± 1.1	4.4 ± 0.1	4.4 ± 0.1	0.570
Body mass (g)		9.7 ± 0.6	8.6 ± 0.7	7.9 ± 0.7	0.187
Body mass index (g cm^-1^)		0.48 ± 0.03	0.44 ± 0.04	0.44 ± 0.03	0.695
Coelomic fat body (mg)		49.2 ± 17.1^a^	5.7 ± 2.9^b^	1.5 ± 0.6^b^	<0.0001
Carcass organic matter	concentration (mg g^-1^)	820 ± 5^a^	784 ± 6^b^	789 ± 5^b^	0.0008
	total (mg)	1298 ± 126	1128 ± 135	1171 ± 128	0.409
Carcass nitrogen	concentration (mg g^-1^)	106 ± 1^a^	111 ± 1^a^	119 ± 2^b^	<0.0001
	total (mg)	168 ± 16	160 ± 20	175 ± 17	0.643
Carcass energy	concentration (kJ g^-1^)	19.2 ± 0.2^a^	17.6 ± 0.3^b^	17.1 ± 0.3^b^	0.0002
	total (kJ)	30.4 ± 3.0	25.6 ± 3.4	25.7 ± 3.1	0.275
Carcass energy/nitrogen		181.2 ± 2.6^a^	159.4 ± 2.7^b^	145.0 ± 2.9^c^	<0.0001
Liver	hepatosomatic index, HSI	14.9 ± 1.0^a^	19.1 ± 1.4^b^	20.2 ± 0.9^b^	0.0081
	glycogen (μmol g^-1^)	2888 ± 95^a^	3281 ± 77^b^	3246 ± 120^b^	0.0309
	glycogen content (μmol)	911 ± 55^a^	1170 ± 103^ab^	1344 ± 115^b^	0.0371
	glycogen richness (μmol g^-1^ frog)	426 ± 23^a^	630 ± 54^b^	675 ± 41^b^	0.0005
	glucose (μmol g^-1^)	3.5 ± 0.4^a^	8.2 ± 1.6^b^	13.0 ± 3.3^b^	0.0010
Muscle	mass (mg mm^-1^ tibiofibula)	1.34 ± 0.06^a^	0.83 ± 0.04^b^	0.93 ± 0.06^b^	<0.0001
	glycogen (μmol g^-1^)	339 ± 14^a^	433 ± 26^b^	366 ± 17^ab^	0.0278
	glucose (μmol g^-1^)	7.5 ± 1.4	4.9 ± 0.4	6.8 ± 1.3	0.342
N		8	10	14	

Values are mean ± SEM. Concentrations of metabolites and energy are expressed per g dry tissue. Glycogen is expressed as glucosyl units. Groups not sharing a common superscripted letter were statistically distinguishable (*P*<0.05).

Winter conditioning also induced changes in skeletal muscles (Table 2). Gastrocnemius mass was 30–38% less in HYD and DHYD frogs than in late-summer frogs. Gracilis of winter-conditioned frogs had relatively high glycogen levels, although the concentration in DHYD frogs was not significantly different from that in late-summer frogs. Glucose concentration in gracilis did not vary among the three groups.

We examined winter-conditioning effects on plasma levels of select metabolites by comparing values between late-summer frogs and HYD frogs (Table 3). There were no differences with glucose or lactate, but uremic levels in HYD frogs, which reached 86 μmol ml^-1^, were elevated 8-fold. Plasma osmolality of late-summer frogs was 225 ± 7 mOsmol kg^-1^, of which ~15 units derived from the collective activities of glucose, lactate, and urea (Table 3). The substantially higher value for HYD frogs, 374 ± 7 mOsmol kg^-1^, was only partly due to their markedly higher uremia, as the increase in urea concentration (75 units) accounted for only half the increment (149 units) in total osmolality. Therefore, HYD frogs apparently co-accumulated with urea a comparable amount of some unidentified solute(s). Plasma protein concentrations in late-summer frogs and HYD frogs were indistinguishable.

**Table 3:** Plasma variables of *R*. *sylvatica* sampled before or after winter conditioning, with or without moisture restriction.

	Late summer	Hydrated	Dehydrated	P
Protein (mg ml^-1^)	28.4 ± 0.8^a^	27.8 ± 2.3^a^	38.9 ± 0.9^b^	<0.0001
Glucose (μmol ml^-1^)	2.4 ± 0.2^a^	5.2 ± 2.2^ab^	6.9 ± 1.7^b^	0.001
Lactate (μmol ml^-1^)	2.6 ± 0.2^a^	2.7 ± 0.3^a^	4.2 ± 0.3^b^	0.002
Urea (μmol ml^-1^)	10.6 ± 1.6^a^	85.5 ± 2.9^b^	186.9 ± 12.4^c^	<0.0001
Osmolality (mOsmol kg^-1^)	225 ± 7^a^	374 ± 7^b^	526 ± 17^c^	<0.0001
N	8	10	14	

Values are mean ± SEM. Groups not sharing a common superscripted letter were statistically distinguishable (*P*<0.05).

Comparing values for HYD and DHYD frogs revealed several effects of dehydration during winter conditioning. Expectedly, DHYD frogs had higher plasma concentrations of all metabolites, but, because the dehydration was rather modest, for most metabolites the difference was minor or, in the case of glucose, statistically non-significant (Table 3). On the other hand, uremia in DHYD frogs, 186.9 ± 12.4 μmol ml^-1^, was inordinately high, being ~88 units above the level resulting solely from dehydration (i.e., an increase from 86 to 99 μmol ml^-1^ was expected, assuming an overall 14% reduction in water concentration). Thus, during winter conditioning DHYD frogs accrued substantially more urea than HYD frogs primarily by increasing ureagenesis. Plasma osmolality of DHYD frogs, 526 ± 17 mOsmol kg^-1^, was 152 units higher than that of HYD frogs (Table 3). Of this increment, an estimated 61 units were due to water removal, with the remainder, 91 units, nearly matching the increase in urea, 88 μmol ml^-1^; this congruence implies that, during winter conditioning, DHYD frogs accumulated no solute other than those accruing in HYD frogs.

Analysis of plasma pooled from individuals comprising the late-summer, HYD, and DHYD groups showed marked differences in amino acid profiles. Although sensitivity, accuracy, and technical reproducibility with ion-exchange column chromatography is quite good [12], we used a conservative threshold of 20% to discern differences in group values for the 34 amino-containing compounds we quantified (Table 4). All but six compounds differed in abundance between late-summer frogs and HYD frogs, most (75%) of the dynamic ones having increased during winter conditioning. Increases exceeding 3-fold were seen in six compounds including threonine, whose concentration was more than 14-fold higher in HYD frogs than in late-summer frogs. However, HYD frogs also showed marked decreases in certain amino acids, including histidine, glycine, and β-alanine, which all fell by at least 50%. Effect of dehydration on the amino acid pool was assessed by comparing values for HYD and DHYD groups. With dehydration, eighteen compounds increased (by as much as 136%), three decreased, and thirteen were unchanged. Most of the compounds that became more abundant in DHYD frogs were also ones that increased in HYD frogs over late-summer frogs, although this group also included the three aforementioned amino acids that decreased most profoundly during winter conditioning.

**Table 4:** Concentrations of amino acids in plasma (nmol ml^-1^) of *R*. *sylvatica* sampled before or after winter conditioning, with or without moisture restriction.

Amino acid	Late summer	Hydrated	Dehydrated
Threonine	113	1605*	1658
Cystathionine	2	18*	23†
3-Aminoisobutyric acid	6	25*	35†
Alanine	334	1348*	1414
Aspartic acid	21	86*	144†
2-Aminoadipic acid	2	6*	11†
2-Aminobutyric acid	10	26*	32†
Serine	92	248*	241
Asparagine	135	354*	496†
Citrulline	29	73*	49†
Valine	107	264*	249
Isoleucine	70	164*	142
Leucine	117	266*	234
3-Methylhistidine	8	18*	28†
Glutamic acid	63	119*	188†
Hydroxyproline	17	30*	40†
Taurine	44	69*	108†
Ornithine	59	91*	96
Phosphoethanolamine	28	37*	23†
Proline	23	30*	39†
Homocystine	1	1	3†
Lysine	135	158	130
Arginine	24	28	22
Methionine	18	18	25†
Cystine	4	4	6†
Tyrosine	44	44	51
4-Aminobutyric acid (GABA)	7	6	5
Glutamine	158	124*	112
1-Methylhistidine	4	3*	4†
Phenylalanine	51	34*	31
Tryptophan	10	6*	1†
Histidine	180	54*	91†
Glycine	286	74*	102†
β-Alanine	151	36*	84†
Total	2355	5466*	5917†

Values were determined for a single pool of plasma derived from *N* = 8–11 frogs in each group. Values for hydrated frogs marked with an asterisk (*) differed from corresponding value for late-summer frogs by ≥ 20%. Values for dehydrated frogs marked with a dagger (†) differed from corresponding value for hydrated frogs by ≥ 20%.

## Responses to Freezing

**Fig 1:**
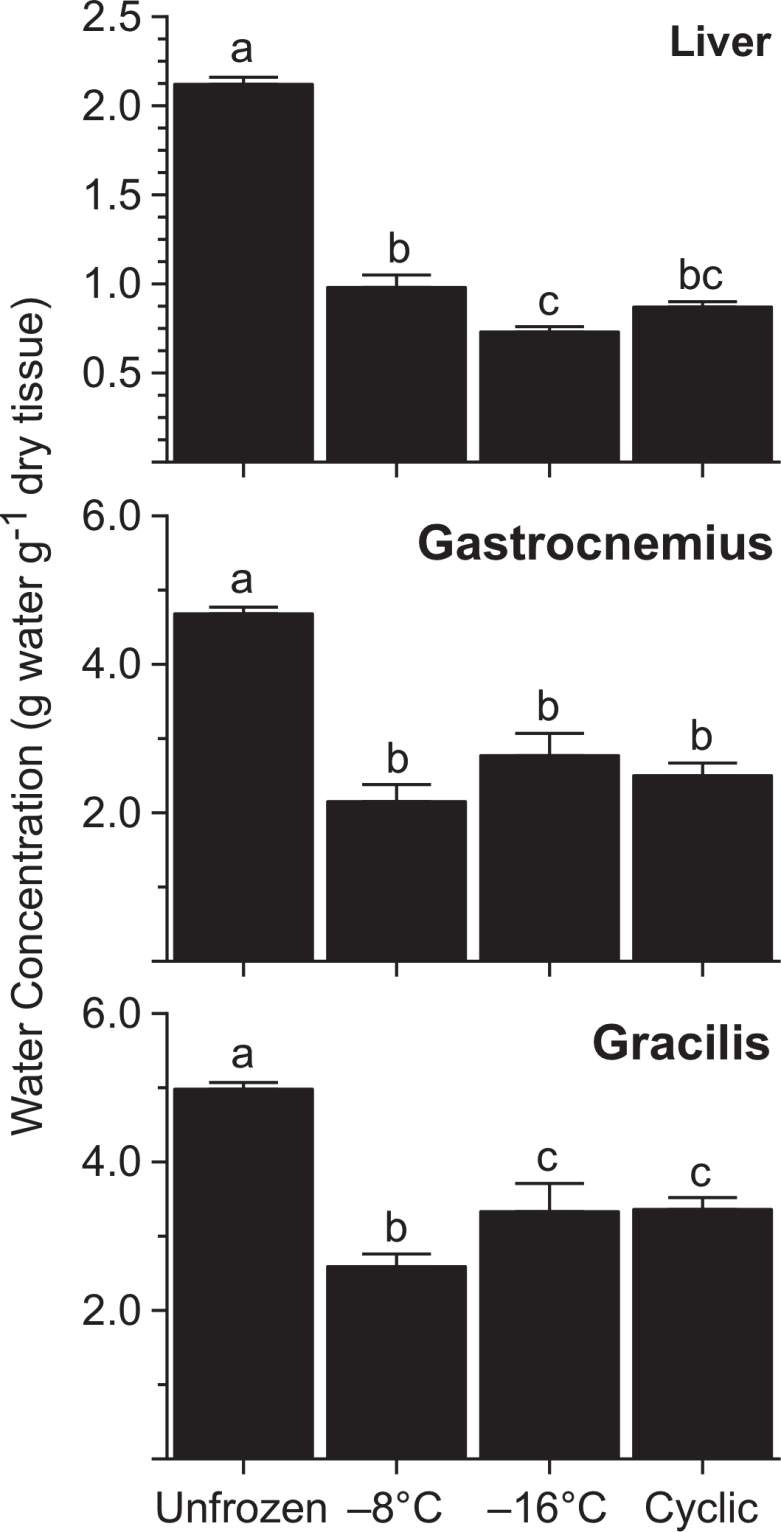
Water concentration in tissues of *R*. *sylvatica* experimentally frozen to −8 or −16°C, or subjected to multiple cycles of freezing and thawing, relative to that in unfrozen controls. Values (mean ± SEM; *N* = 6–7) not sharing a common letter were statistically distinguishable (*P*<0.05).

Frogs experimentally frozen to -8 or -16°C were icy, rigid, and inanimate. We observed granules and thin plates of ice beneath the skin, along organs within the coelom, and among muscle fibers of the hind limbs, which appeared desiccated and ruddy. The liver also appeared shrunken and desiccated. Water concentration in organs varied strongly (*P*<0.0001, all cases) among groups, attesting that tissues dehydrated with freezing; water loss was up to 66% in liver, 41–54% in gastrocnemius, and 33–48% in gracilis (Fig. 1). Among the groups of frozen frogs, there were relatively minor, albeit statistically significant, differences in tissue hydration.

**Fig 2:**
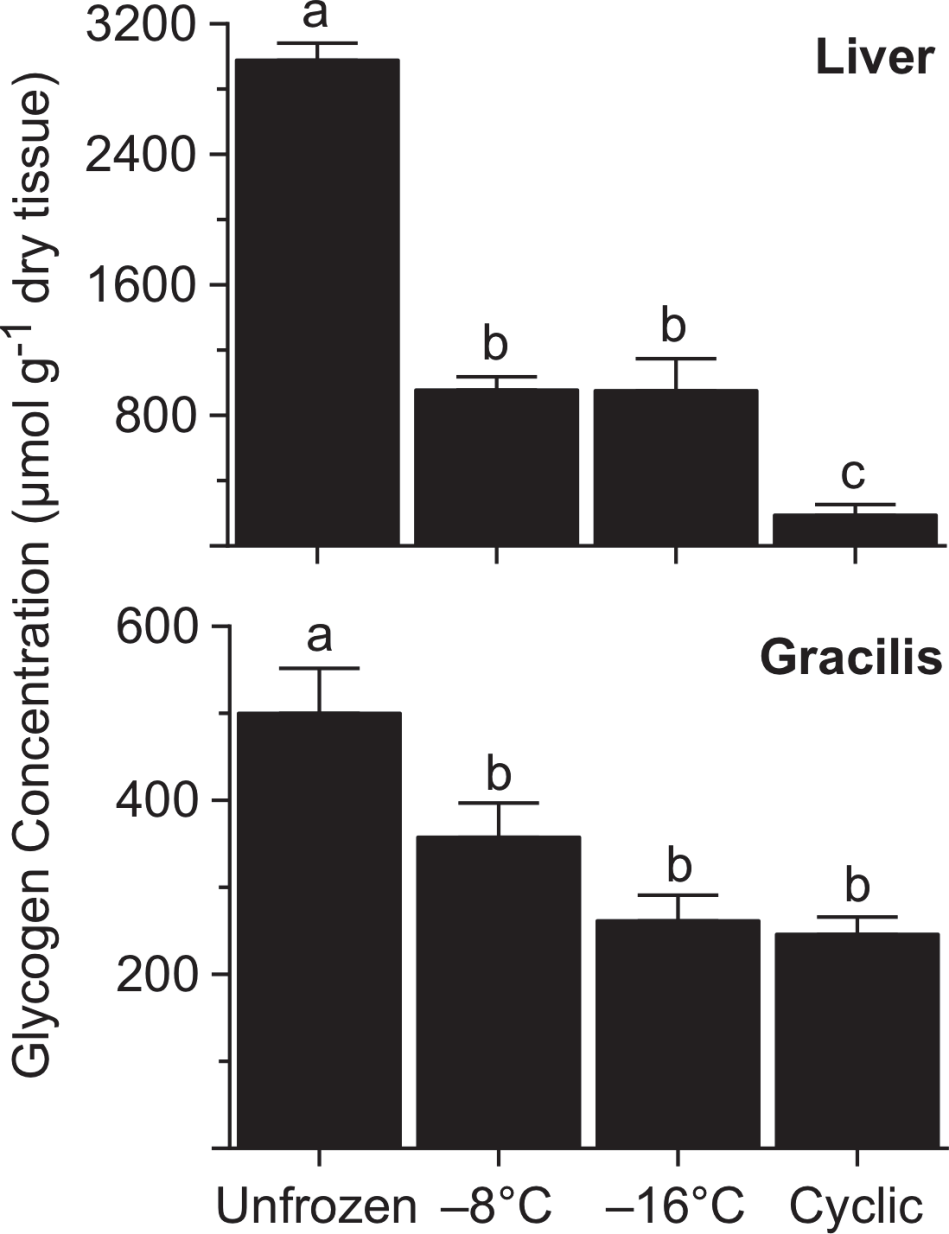
Glycogen concentration in tissues of *R*. *sylvatica* experimentally frozen to −8 or −16°C, or subjected to multiple cycles of freezing and thawing, relative to that in unfrozen controls. Values (mean ± SEM; *N* = 6–7) not sharing a common letter were statistically distinguishable (*P*<0.05).

The conspicuous contraction of the liver in frozen frogs also stemmed from reduction in the organ’s dry mass, as HSI in these frogs was markedly lower (*F* = 17.9, *P*<0.0001) than that of unfrozen (control) frogs, 16.8 ± 1.7%. HSI values of frogs in the -8 and -16°C groups (9.6 ± 0.9% and 9.7 ± 0.7%, respectively) were indistinguishable, but greater than those of the cyclic-freeze group, 6.7 ± 0.3%. Frogs frozen to -8 or -16°C had hepatic glycogen levels only ~32% of those found in unfrozen frogs, 2978 ± 105 μmol g^-1^ dry tissue (Fig. 2), indicating that much of the drop in the liver’s dry mass was due to freezing-induced glycogenolysis. Moreover, glycogen concentration in livers of the cyclic-freeze frogs was lower still, being just 6% of the value for unfrozen frogs. Freezing also triggered glycogenolysis in skeletal muscle, the mean glycogen concentrations in gracilis being 30–50% below the value for unfrozen frogs (Fig. 2).

Difficulty collecting blood from small, frozen specimens prevented us from analyzing plasma metabolite levels for most frogs. However, values obtained for the unfrozen frogs (*N* = 7) are useful for establishing baseline metabolite levels in the winter-conditioned frogs used in this experiment. Plasma levels of glucose (8.8 ± 2.0 μmol ml^-1^), lactate (1.1 ± 0.2 μmol ml^-1^), and urea (74.4 ± 6.4 μmol ml^-1^), and plasma osmolality (354 ± 11 mOsmol kg^-1^), appeared similar to values found in HYD frogs at the end of winter conditioning (Table 3).

**Fig 3:**
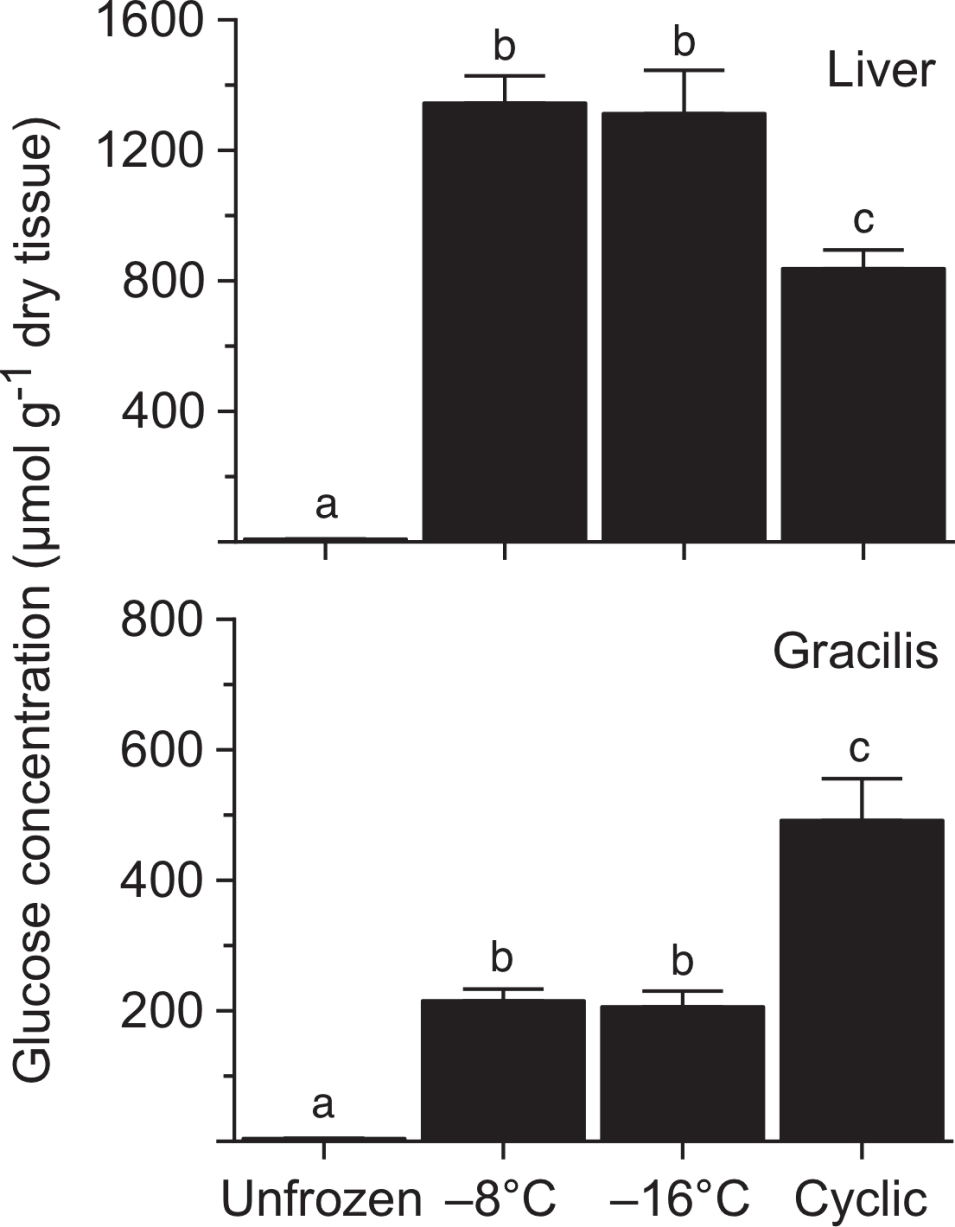
Glucose concentration in tissues of *R*. *sylvatica* experimentally frozen to −8 or −16°C, or subjected to multiple cycles of freezing and thawing, relative to that in unfrozen controls. Values (mean ± SEM; *N* = 7) not sharing a common letter were statistically distinguishable (*P*<0.05).

Concentration of glucose in liver increased dramatically during freezing (*F* = 784.3, *P*<0.0001) with levels in frogs frozen to -8 or -16°C exceeding 1300 μmol g^-1^ dry tissue (Fig. 3). Levels in frogs subjected to cyclic freezing were not quite as high, but still 100-fold greater than those in unfrozen frogs, 8.1 ± 0.8 μmol g^-1^ dry tissue. Muscle also showed a robust increase (*F* = 358.7, *P*<0.0001) in glucose with freezing, but the pattern of variation among groups differed from that seen with liver: glucose concentration in gracilis of the cyclic-freeze frogs was 2.3-fold higher than that found in frogs frozen to -8 or -16°C.

**Fig 4:**
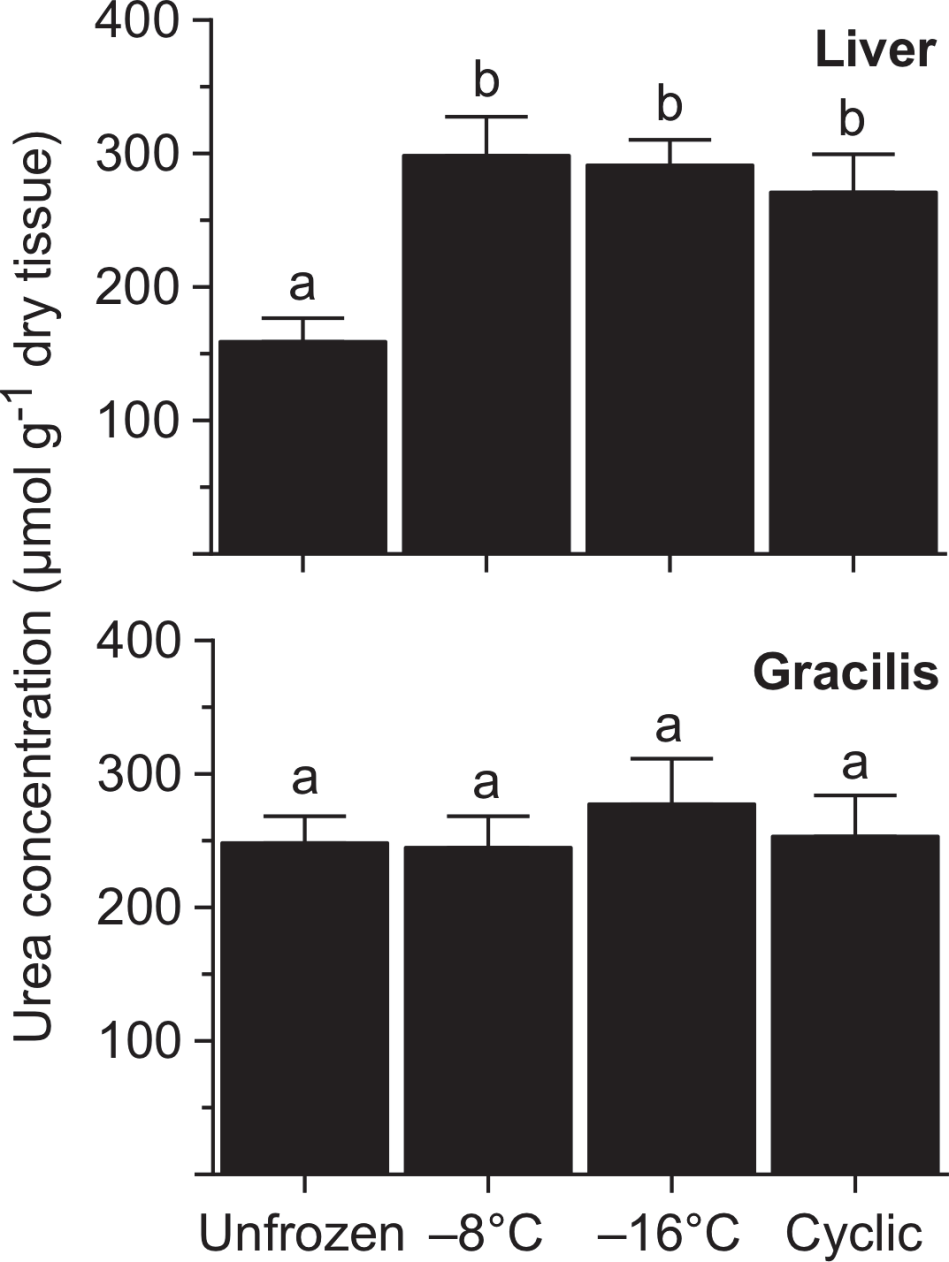
Urea concentration in tissues of *R*. *sylvatica* experimentally frozen to −8 or −16°C, or subjected to multiple cycles of freezing and thawing, relative to that in unfrozen controls. Values (mean ± SEM; *N* = 7) not sharing a common letter were statistically distinguishable (*P*<0.05).

Urea concentration in liver increased (*F* = 7.2, *P* = 0.0013) with freezing, as the level in every group of frozen frogs was about two-fold greater than that in unfrozen frogs (Fig. 4). In contrast, urea concentration in muscle did not vary (*F* = 0.3, *P* = 0.84) among groups.

**Fig 5:**
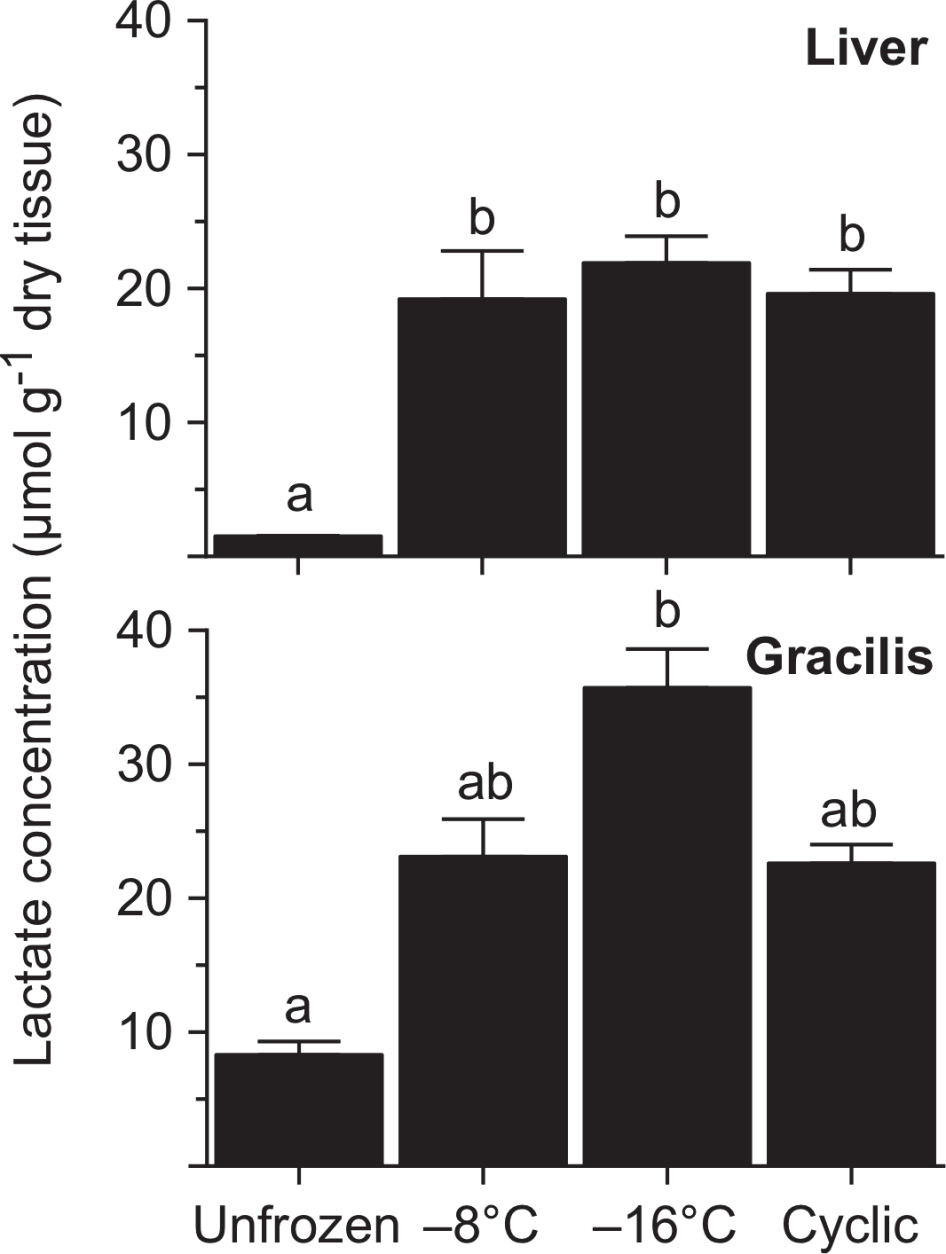
Lactate concentration in tissues of *R*. *sylvatica* experimentally frozen to −8 or −16°C, or subjected to multiple cycles of freezing and thawing, relative to that in unfrozen controls. Values (mean ± SEM; *N* = 7) not sharing a common letter were statistically distinguishable (*P*<0.05).

Lactate concentration in liver was relatively low (1.5 ± 0.04 μmol g^-1^ dry tissue) in unfrozen frogs, but markedly higher (*F* = 158.7, *P*<0.0001) in frozen frogs. The particular freezing treatment used had no influence on the amount of lactate accumulating in liver, although a significant increase in muscle lactate occurred only in frogs frozen to -16°C (*F* = 20.6, *P* = 0.0001; Fig. 5).

## Survival of Freezing

In our freeze tolerance trial, all five of the frogs subjected to cyclic freeze/thaw episodes and ultimately cooled to -8°C exhibited normal behaviors when first examined, ~30 h after thawing was initiated. They remained healthy during an ensuing 7-week period of monitoring. SBM of these frogs determined immediately before freezing (2.82 ± 0.17 g) and after thawing (2.73 ± 0.20 g) did not differ (paired *t*-test: *t* = 1.85, *P* = 0.139), indicating that they did not lose body water or substantially catabolize tissue during the trial.

## Discussion

Wood frogs from populations near the northern limit of the species’ range are adapted to survive corporal freezing at temperatures well below those that can be tolerated by conspecifics from lower latitudes [6, 10]. Our aim in the present investigation was to further explore the unique mechanisms by which northern frogs physiologically prepare for the extreme and lengthy subarctic winter, and to elucidate how their cryoprotectant systems have been enhanced to permit survival of freezing at such remarkably low temperatures.

## Preparations for Overwintering

### Energetic Transitions

In subarctic populations of *R*. *sylvatica*, physiological preparation for winter begets a marked increase in the hepatic glycogen store (30–50% in the present investigation; 37% in our earlier study; [6]), which contributes importantly to surviving the ensuing winter. This process is facilitated by seasonally high levels of glycogen synthase in hepatocytes [13] and probably draws heavily on any assimilated nutrients. However, because feeding is limited during this time of decreasing environmental temperature, frogs attain the massive glycogen depot primarily by accelerating gluconeogenesis, which potentially explains the uncharacteristically high glucose levels in liver and plasma of our winter-conditioned frogs.

Gluconeogenesis in the pre-hibernal period apparently is driven by the breakdown of lipids to suitable precursors. Our frogs, like those examined previously [6], catabolized virtually their entire coelomic fat body during winter conditioning. The glycerol moiety of triglyceride could be a major precursor in gluconeogenesis and glycogen synthesis, even more so than preformed glucose [14]. In addition, fat catabolism could yield ketones, which, by fueling metabolism in muscle, brain, and (preferentially) heart, would augment glycogen storage by reducing cells’ need to oxidize hexose. Moreover, converting lipids to a fermentable fuel before winter’s onset seems advantageous given that fatty acids cannot be oxidized in anoxic, frozen tissues. Depletion of coelomic fat body (whose size correlates to overall lipid content; [15, 16]), together with enzymatic changes curtailing fat catabolism in winter [17], implies that triglyceride is a relatively unimportant energy substrate in hibernating *R*. *sylvatica*. This contrasts with the case of desert anurans, which heavily draw on lipid stores to fuel metabolism during dormancy [16, 18, 19].

We found that protein catabolism plays an important, previously unrecognized, role in the hibernal preparations of *R*. *sylvatica*. Free amino acids in plasma were 130–150% more abundant in winter-conditioned frogs than in late-summer frogs, the multi-fold increases in various glucogenic amino acids (particularly threonine, alanine, serine, and asparagine) likely augmenting glycogen synthesis in the pre-hibernal period and possibly even supporting maintenance metabolism during winter. At the same time, catabolism of these compounds produced copious urea, which accrues in response to oliguria, reduced rates of glomerular filtration, and reabsorption in the urinary bladder, and is retained in hibernation for use as metabolic depressant [20] and cryoprotectant [7]. Notably, uremia in fully-hydrated, winter-conditioned frogs, both in the present study and in Costanzo et al. [6], increased ten-fold over that found in late-summer frogs. These northern frogs amassed even more urea than accumulates in temperate counterparts undergoing experimental dehydration [21].

We monitored changes in energy balance of frogs during winter conditioning to gain insights into the potential role of starvation in the proteolytic response. Complements of crickets consumed by hydrated and dehydrated frogs contained considerable energy (4.5 ± 0.9 and 5.0 ± 1.2 kJ, respectively), although, overall, these frogs probably expended more energy than they acquired. Their carcasses exhibited reduced concentrations of energy and organic matter, but an apparent increase in nitrogen, which became concentrated in the remaining organic component. However, these changes do not necessarily signify metabolic distress, as some matter and energy were simply transferred from atrophying muscle to glycogen stores in liver, which was excluded from the material being analyzed. Our findings that total carcass energy was not significantly reduced, and that glycogen depots in liver and muscle were enlarged (rather than depleted, a hallmark of starvation; [22]), argue that these frogs were not starving, but rather underwent an adaptive metabolic reorganization during winter conditioning.

### Muscle Atrophy in the Pre-hibernal Period

Mass of the gastrocnemius decreased by 31–38% during winter conditioning, indicating that skeletal muscle is a principal source of the protein consumed in the pre-hibernal period. Costanzo et al. [6] observed a comparable change in that muscle, as well as a 40% drop in protein concentration in gracilis, in frogs from the same population. Atrophy probably also occurs in other muscles, although proteolytic response to altered physiological state can be highly muscle specific [23–25]. Future studies should determine the full extent of the atrophy and, since *R*. *sylvatica* engages in strenuous mating activity upon hibernal emergence, its potentially adverse effect on muscle performance. They should also elucidate mechanisms underlying the differential responses between *R*. *sylvatica* and atrophy-resistant anurans that effectively preserve the mass and mechanical properties of muscles during prolonged hibernation [26] or estivation [27]. Extensive catabolism of muscle tissue in pre-hibernal *R*. *sylvatica* potentially stems from changes in endocrine status, as, for example, protein degradation is accelerated by insulin deficiency [28]. Some ranids exhibit a marked seasonal rhythm in serum insulin, with levels falling sharply at winter’s advent [29], coincident with reduction in the hormone’s receptor population [30]; such changes conceivably could explain the hyperglycemia and depleted fat body in our pre-hibernal frogs. Furthermore, insulin secretion is partly mediated by the neurotransmitter, 5-hydroxytryptamine, which also declines in autumn [31], possibly due to the shortened photoperiod acting on the pineal body. A putative seasonal decline in 5-hydroxytryptamine is consistent with the observed reduction in its precursor, tryptophan, in our winter-conditioned frogs.

### Urea Accrual in Hydrated Frogs

The importance of urea in amphibian physiology is best recognized for its role in defending body water during periods of osmotic stress (see review: [8]). Costanzo and Lee [7] proposed that this compound, acting as both an osmoprotectant and a cryoprotectant, also confers a survival advantage to terrestrially hibernating frogs. In their study, uremia in *R*. *sylvatica* from a cool-temperate population (southern Ohio) varied seasonally from 2 to ~50 μmol ml^-1^, the higher values coinciding with reductions in environmental water potential and body water content. However, a recent study revealed that osmotic stimuli are not essential for frogs to accumulate urea for winter use, as urea reached 29 μmol ml^-1^ in Ohioan *R*. *sylvatica* that remained hydrated during simulated hibernation [6]. Moreover, Alaskan frogs accrue remarkably high levels of urea (86 μmol ml^-1^ herein; 106 μmol ml^-1^ in Costanzo et al. [6]) during winter preparation despite remaining in water balance. This phenomenon may be unique to terrestrial hibernators, and perhaps some (although not all; see [32]) freeze-tolerant species, since ranids that hibernate under water sustain or even reduce initial uremia during cold acclimation [33, 34]. Indeed, even euryhaline and desert-adapted forms commonly maintain a low uremia (e.g., 5–10 μmol ml^-1^) when hydrated [9].

In our pre-hibernal *R*. *sylvatica*, ureagenesis was enhanced by adjustments at the molecular level in liver and skeletal muscle. In hydrated frogs, the pattern of change with winter conditioning in the plasma free amino acid pool, notably an increase in glutamate, alanine, and valine, and a decrease in glutamine, resembles the change in free amino acids occurring in skeletal muscle during estivation of the spadefoot toad, *Scaphiopus couchii* [35]. In our Alaskan *R*. *sylvatica*, an apparent exchange of glutamine for glutamate (via the glutaminase reaction) and marked rise in compounds (alanine, aspartate) that readily transfer amine groups to glutamate are coupled with a coordinated, 70% rise in glutamate dehydrogenase activity in muscle [6]. Altogether, these changes yield an abundance of ammonium ions (from glutamate deamination) that would accelerate ureagenesis in liver [36].

Conceivably, *R*. *sylvatica* could also accelerate ureagenesis during winter conditioning by upregulating the ornithine-urea cycle (OUC) by a process similar to that used by some species of estivating frogs [36–38]. Indeed, hepatic activity of carbamoyl phosphate synthetase 1, a rate-limiting enzyme associated with the OUC, is on the order of that expressed in saline-adapted anurans, and is held at high levels from summer through winter [39]. Furthermore, our present results show that, during winter conditioning, frogs elevated four of the five associated α-amino acids, including the non-proteinogenic compounds, citrulline and ornithine. Whether or not rates of ureagenesis materially increase during the pre-hibernal period requires empirical testing, but, clearly, in these frogs the metabolic infrastructure is well poised to rapidly synthesize urea, even in the face of declining ambient temperatures.

### Urea Accrual in Dehydrated Frogs

Consistent with its terrestrial habits, *R*. *sylvatica* is well adapted to survive dehydration [40], although, in the extreme, frogs may incur ischemic hypoxia and increased production of lactate and/or glucose [21, 41]. Our frogs dehydrated only modestly, and the nominal rise in these metabolites simply reflects the concentrating effect of solvent reduction. By contrast, plasma urea rose disproportionately and dramatically, averaging a remarkable 187 μmol ml^-1^ and even exceeding 0.25 M in one individual. Possibly, Alaskan *R*. *sylvatica* undergoing profound dehydration, or smaller, more dehydration-susceptible individuals, could achieve an even higher uremia. Ability to accumulate urea to high levels varies by taxa and generally correlates with habitat preference [42, 43], although the case with *R*. *sylvatica*, a mesic woodland species, clearly is exceptional. Indeed, the concentrations of urea found in our frogs are comparable to those reported for euryhaline and xeric-adapted forms undergoing estivation or osmotic stress (see Table 1 in Withers and Guppy; [9]).

The markedly higher uremia of dehydrated frogs implies that they catabolized more protein during winter conditioning than did hydrated frogs. This is not apparent from the decrease in gastrocnemius mass (a relatively coarse metric), but is supported by their relatively low energy:nitrogen ratio. Given that nitrogen is retained during winter conditioning, but is shifted from a high-energy molecule (protein) to a low-energy one (urea), protein degradation is manifest in a drop in energy:nitrogen ratio, which in fact was more pronounced in dehydrated frogs (Table 2). Enhanced muscle catabolism in dehydrated frogs is also indicated by their markedly higher plasma levels of two metabolites important to ureagenesis, glutamate and aspartate, as well as 3-methylhistidine, which, being neither metabolized nor reused in protein synthesis, signals degradation of myofibrillar protein [28].

Some xeric-adapted frogs upregulate urea synthesis during osmotic stress in part by catabolizing muscle protein [19, 37], with the attendant deamination releasing carbon skeletons that substantially increase glycogen deposition in liver [44]. The glycogen store in dehydrated *R*. *sylvatica* was not significantly larger than that of hydrated frogs; however, they might have synthesized more glycogen had our experimental dehydration been more protracted or severe. Conceivably, partial dehydration in the pre-hibernal period may benefit these frogs by bolstering both their uremic and glucosic cryoprotectant systems. In temperate regions, dehydration seems likely because the water potential of soil is particularly low in autumn [7] and drier, upland sites are preferred for hibernation [45]. In Interior Alaska, *R*. *sylvatica* hibernates closer to water bodies [10] but nevertheless favors relatively dry microsites for overwintering [46].

### Osmolytes in Winter-Conditioned Frogs

Plasma osmolality in winter-conditioned frogs were within the lower range of those achieved by hyperuremic, estivating anurans [9, 18]. In many amphibians, osmotic activity of the blood is accounted for chiefly by the concentrations of principal ions and urea; however, a considerable osmotic gap (>50 mOsmol kg^-1^) can occur in some species, including salt-adaptable and urea-accumulating estivators [9]. Similarly, in our Alaskan *R*. *sylvatica* (and those examined by Costanzo et al. [6]), plasma osmolality was about 75 mOsmol kg^-1^ higher than expected, indicating that some extraordinary solute(s) co-accumulated with urea during winter conditioning; in contrast, conspecifics from a more temperate population, which do not accumulate as much urea, have no osmotic gap [47]. Future studies directed at ascertaining the identity and source of the unique osmolyte(s) might profitably examine methylamines, a class of compounds known to counteract the perturbing effects of high urea on macromolecular structure and function [48]. Anurans do not commonly accrue these compounds to significant levels in salt adaptation or estivation [9, 43, 49], although both trimethylamine *N*-oxide (TMAO) and glycerophosphorylcholine (GPC) reportedly vary seasonally in *R*. *sylvatica* from the Great Lakes Region of North America, being most abundant in the fall and winter [50]. TMAO protects macromolecules against high salt concentrations [48, 51, 52] as well as freeze/thaw injury [53], and thereby may serve a cryoprotective role.

In some organisms, winter conditioning or cold acclimation increases the size of the pool of free amino acids [54]. In the present study, this response had only a minor impact on total osmotic activity, but probably nevertheless contributed to winter survival. Some amino acids serve as fermentable substrates under hypoxic, frozen conditions [54]. Some, including alanine, proline, and glutamic acid, which increased with winter conditioning in our *R*. *sylvatica*, effectively stabilize proteins and improve cell survival under freeze/thaw stress [53, 55, 56].

## Freezing Responses

### Organ Dehydration

In *R*. *sylvatica*, corporal freezing is accompanied by an extensive, reversible dehydration of the organs. Physicochemical forces governing the process are not fully understood, although it is clear that organs dehydrate to variable degrees as freezing proceeds, and the translocated water ultimately freezes within the lymph sacs, subdermal spaces, and coelom [57]. Our present findings indicate that severity of freezing exposure strongly influences the magnitude of the response. In Alaskan frogs frozen to −2.5°C, dehydration was a modest 27% in gracilis and 29% in gastrocnemius [6], whereas, in our frogs frozen to −8 or −16°C, the same muscles dehydrated by up to 48% and 54%, respectively. Furthermore, whereas livers of our frogs dehydrated extensively (54–66%), frogs frozen to −2.5°C had livers that actually hydrated, apparently by liberating more water through glycogenolysis than could be removed during freezing. Limited dehydration of any organ not only reduces mechanical damage to the tissue’s architecture but also parlays cryoprotection by concentrating osmolytes in a reduced solvent volume [57]. The latter effect can be considerable: glucose and urea jointly attained up to 2.1 M in liver and 0.2 M in muscle due to dehydration alone (Table 5), and these levels would be even higher when freeze concentration is considered.

**Table 5:** Concentrations of two cryoprotectants within liver and skeletal muscle of *R*. *sylvatica* subjected to different experimental freezing regimes.

	Liver				Gracilis			
	Unfrozen	–8°C	–16°C	Cyclic	Unfrozen	–8°C	–16°C	Cyclic
Glucose	4	1393	1760	941	1	83	64	146
Urea	75	307	388	305	50	94	86	75
Glucose + urea	79	1700	2148	1245	51	177	150	221

Values, expressed as μmol ml^-1^ tissue fluid, were computed from mean water and solute concentrations and thus incorporate the effect of organ dehydration during freezing. Actual cryoprotectant levels in frozen tissues would be considerably higher due to freeze-concentration of the solution remaining within them.

### Glycemic Response

Freezing survival of *R*. *sylvatica* critically depends on the capacity to mobilize sufficient amounts of the cryoprotectant glucose during the early stages of freezing. In liver, glycogen is rapidly degraded to glucose, which must be distributed to tissues throughout the body before freezing progresses to the point that circulation ceases. Biochemical control of this glycogenolytic response has been studied extensively in *R*. *sylvatica* [54], including the northern phenotype here under investigation [58]. Experimental freezing of Alaskan frogs to −2.5°C caused their hepatic glycogen concentration to fall by 39% and their glycemic level to rise to ~220 μmol ml^-1^ [6]. Because they can encounter substantially lower temperatures in hibernation (e.g., −9 to −18°C; [10]), we hypothesized that these frogs would synthesize additional cryoprotectant under more extreme conditions. Clearly, this was the case: liver dry mass fell precipitously, and its glycogen concentration was reduced by two-thirds in frogs directly frozen to −8 or −16°C, and by nearly 95% in frogs subjected to a cyclic freeze/thaw regimen. That glycogen turnover was independent of endpoint temperature, −8 or −16°C, implies that glucose synthesis had ceased at some temperature above −8°C. Thus, the enhanced glycogenolytic response in our frogs (as compared to that in frogs frozen 48 h to −2.5°C; [6]) may be more a consequence of the longer freezing bout than the lower temperatures to which they were exposed.

Consistent with this enhanced response, glucose concentration in livers of our frogs was 80–85% greater than that found in sympatric frogs frozen to −2.5°C [6]. However, no such increase occurred in skeletal muscle, indicating that the distribution of cryoprotectant to (and/or uptake by) peripheral tissues is curtailed at a relatively high body temperature. The question of whether skeletal muscle and other tissues endogenously synthesize glucose remains open. In *R*. *sylvatica*, corporal freezing reportedly does [59] or does not [60] reduce glycogen concentration in skeletal muscle. In the present study, levels of glycogen in gracilis fell by 30–50% with freezing, but the cryobiological implications of this result are equivocal. Assuming that *R*. *sylvatica* muscle has the enzyme, glucose-6-phosphatase, needed to dephosphorylate glucose-6-phosphate (see [60]), it seems doubtful that the small amount of glucose produced (e.g., up to 3 μmol glucosyl units by a 70-mg gracilis) could contribute substantially to colligative cryoprotection. It could, however, help fuel glycolysis under the prevailing hypoxic conditions.

### Uremic Response

Urea concentration in liver rises in Alaskan (and Ohioan) *R*. *sylvatica* frozen to −2.5°C, suggesting that, as with glucose, hepatic synthesis of this agent is further stimulated by somatic freezing [6]. Accordingly, the concentration nearly doubled during freezing in the present study. However, the cryoprotective benefit is limited because urea levels do not markedly increase with freezing in skeletal muscle (present study) or other organs [6, 7, 61], nor even in the blood [6, 61, 62]. Urea’s export from liver potentially is hampered by a loss of transporters, although in other tissues the transporter population is maintained during freezing [63]. It is also possible that urea efflux from hepatocytes is inhibited by glucose, just as high urea competitively inhibits the transport of glucose [64]. Additional research will be needed to determine why urea synthesized during freezing remains sequestered within the liver.

### Urea and Glucose in Frozen Tissues

*R*. *sylvatica* accumulates urea primarily in anticipation of freezing and glucose after freezing has begun. Both agents colligatively limit ice formation and cellular dehydration, but each also provides benefits such as antioxidation, metabolic regulation, glycolytic fuel supply, ischemic injury prevention, membrane stabilization, and protein protection and renaturation [47, 54]. Although high levels of urea are well tolerated [43], sustained hyperglycemia potentially could cause glycation of lipids and proteins, among other problems. The on-demand feature of this glucosic cryoprotectant system reduces that risk, but the need to rapidly synthesize and distribute glucose before vascular conduits freeze is never fully met, and, consequently, the glucose concentrations ultimately realized by peripheral organs are relatively low [54]. By contrast, urea is more uniformly distributed among tissues, but the amount accrued is influenced by vagaries of environmental temperature and moisture availability (Table 2; see also [7]). Thus, frozen tissues can vary markedly in the particular mix of the agents they contain. For example, in frogs directly frozen to −8 or −16°C, glucose predominated in liver, representing 82% of total cryoprotectant, whereas urea accounted for 53–57% of all cryoprotectant in skeletal muscle (Table 5). Future study should determine whether the protective efficacy of each agent varies in accordance with its predominance within organs.

### Freezing Stress at Low Temperature

Corporal freezing typically results in cardiac arrest and circulatory failure, and, hence, ischemic hypoxia. Protracted or severe freezing necessitates an even greater reliance on anaerobic glycolysis [65] and, accordingly, muscle concentrations of lactate were about twice as high in our frogs than in others frozen to −2.5°C [6]. However, congruence in lactate levels between frogs frozen to −8 or −16°C suggests that full reliance on glycolysis for energy production occurs at some temperature above −8°C. Frogs exposed to repeated cycles of freezing apparently did not accrue additional lactate, probably because some of this metabolite was oxidized or reconverted to glucose during the brief intervals of thawing.

### Responses to Cyclic Freezing

In the present study, Alaskan *R*. *sylvatica* readily recovered from being frozen several times to −4°C before being cooled to −8°C over the course of 19 d. Although freezing adaptations are conventionally studied in subjects given a single bout of experimental freezing, natural fluctuations in ambient temperature can expose organisms to successive cycles of freezing and thawing that potentially could alter their freezing responses and susceptibility to freezing injury. For example, freezing multiple times in quick succession could cause reactive-oxygen species, generated during re-oxygenation of tissues upon thawing, to accrue to levels that overtax anti-oxidation systems [66]. In insects and frogs, serial freeze-thaw cycles cause energy stress and tissue damage, and can increase mortality [65, 67–70].

On the other hand, intermittent thawing could be beneficial, as, for example, it enables organisms to restore homeostatic mechanisms and mitigate or repair damage accruing during prolonged freezing (e.g., [71]). It apparently enhances the cryoprotectant response in *R*. *sylvatica*, as frogs subjected to multiple freeze/thaw cycles catabolized nearly their entire glycogen depot, probably by resuming glycogenolysis during bouts of refreezing. This superproduction of glucose, coupled with restored circulation during thawing intervals, allowed them to achieve uncharacteristically high levels of cryoprotectant in leg muscle (Table 5) and probably other peripheral tissues as well. In Alaskan frogs, accrual of glucose with successive freezing bouts is enhanced because mobilized sugar persists in tissues long after thawing [6], perhaps owing to inhibitory effects of high urea on the liver’s re-uptake of glucose [64] and/or glucose’s re-conversion to glycogen [61]. By contrast, in frogs of temperate populations, tissue glucose levels are greatly diminished within hours or a few days of thawing [59, 72].

Having found that Interior Alaskan *R*. *sylvatica* experience a series of shallow freeze-thaw cycles in early October, just prior to a deeper, more prolonged freezing exposure, Larson et al. [10] argued that such cycles explained the exceptionally high glucose levels measured in frozen frogs sampled from outdoor enclosures in mid-December, and were key to the extreme freeze tolerance exhibited by subarctic frogs. However, frogs from that region readily survive direct, experimental freezing to temperatures at least as low as −16°C [6] and, furthermore, our present results attest that they can achieve extraordinarily high concentrations of glucose during a single freezing bout of sufficient duration and severity. Indeed, the mean glucose concentration in liver of frogs directly frozen to −16°C was 743 μmol g^-1^ fresh tissue, only slightly less than that (788 μmol g^-1^ fresh tissue) determined for frogs experiencing repeated freezes in the field [10]. Nevertheless, the potentially higher glucose levels—especially in peripheral tissues—achieved by frogs undergoing a series of shallow freeze/thaw episodes could improve survival of protracted or severe freezing exposures, including those involving rapid cooling. In early winter, before an insulating snowpack develops, cooling rates of Alaskan frogs in their natural hibernacula can be remarkably high (up to 1.60°C h^-1^; [10]), even exceeding those causing mortality in Ohioan conspecifics [11]. Nevertheless, by incrementally preloading their cells with cryoprotectant these frogs can overcome the problem that rapid freezing hampers production and distribution of glucose to non-hepatic tissues.
