# Correction: Cryoprotectants and Extreme Freeze Tolerance in a Subarctic Population of the Wood Frog

**DOI:** 10.1371/journal.pone.0124029

**Published:** 2015-04-02

**Authors:** The PLOS ONE Staff

Due to a typesetting error, the footnotes for Table 5 do not appear beneath the table. Instead, the footnotes are erroneously included as paragraph two in the Organ Dehydration subsection of the Freezing Responses section of the Discussion.

Please view the complete, correct Table 5 below.

**Table 5:** Concentrations of two cryoprotectants within liver and skeletal muscle of *R. sylvatica* subjected to different experimental freezing regimes.

	**Liver**				**Gracilis**			
	Unfrozen	–8°C	–16°C	Cyclic	Unfrozen	–8°C	–16°C	Cyclic
Glucose	4	1393	1760	941	1	83	64	146
Urea	75	307	388	305	50	94	86	75
Glucose + urea	79	1700	2148	1245	51	177	150	221

Values, expressed as μmol ml^-1^ tissue fluid, were computed from mean water and solute concentrations and thus incorporate the effect of organ dehydration during freezing. Actual cryoprotectant levels in frozen tissues would be considerably higher due to freeze-concentration of the solution remaining within them.
